# Fano resonances in bilayer graphene superlattices

**DOI:** 10.1038/s41598-017-16838-9

**Published:** 2017-12-01

**Authors:** J. A. Briones-Torres, I. Rodríguez-Vargas

**Affiliations:** Unidad Académica de Física, Universidad Autónoma de Zacatecas, Calzada Solidaridad Esquina Con Paseo La Bufa S/N, 98060 Zacatecas, Zac. Mexico

## Abstract

In this work, we address the ubiquitous phenomenon of Fano resonances in bilayer graphene. We consider that this phenomenon is as exotic as other phenomena in graphene because it can arise without an external extended states source or elaborate nano designs. However, there are not theoretical and/or experimental studies that report the impact of Fano resonances on the transport properties. Here, we carry out a systematic assessment of the contribution of the Fano resonances on the transport properties of bilayer graphene superlattices. Specifically, we find that by changing the number of periods, adjusting the barriers height as well as modifying the barriers and wells width it is possible to identify the contribution of Fano resonances on the conductance. Particularly, the coupling of Fano resonances with the intrinsic minibands of the superlattice gives rise to specific and identifiable changes in the conductance. Moreover, by reducing the angular range for the computation of the transport properties it is possible to obtain conductance curves with line-shapes quite similar to the Fano profile and the coupling profile between Fano resonance and miniband states. In fact, these conductance features could serve as unequivocal characteristic of the existence of Fano resonances in bilayer graphene.

## Introduction

Resonances represent an ubiquitous physical phenomenon, since we can find them in very diverse fields, and from the classical world to the quantum world. Resonances are also fundamental characteristics that provide valuable information and help to understand physical systems. The archetypal resonance is the so-called Breit-Wigner resonance, which has as fundamental characteristic a Lorentzian line-shape. However, from the very beginning of atomic physics a resonance with asymmetrical line-shape was challenging^1^, because it did not obey the usual models. The pioneering work of Ugo Fano about auto-ionizing states in atoms came to solve the mystery of the asymmetrical resonance^2,3^. Fano suggested a simple model based on the constructive and destructive interference of the propagating and discrete states. That model gave place to what now is known as the Fano profile. This profile is characterized by a rather simple mathematical expression, *σ* = (*ε* + *q*)^2^/(*ε* ^2^ + 1). This expression has a phenomenological parameter *q* that gives the profile shape and a reduced energy *ε* = (*E* − *E*_*R*_)/Γ that bears information about the peak position *E*_*R*_ and the width of the resonance Γ. Furthermore, according to the value of *q* we can find different types of resonances: *q* = 1 is a Fano resonance, *q* = 0 an antiresonance, and for large values of *q* (*q* → ∞) a typical Breit-Wigner resonance. The simplicity and elegance of this formula together with the explosion, continuous refinement and sophistication of spectroscopic techniques make of Fano resonances a preponderant phenomenon in science and engineering^4^. In fact, Fano resonances can be found in many fields of physics^5–9^ and with many potential applications^4,10,11^. In most cases, this special type of resonances arise in systems in which continuum states are provided by a light source and discrete states come from size effects. However, it is also possible to find Fano resonances in quantum transport by special nano designs that provide extended and discrete states simultaneously^7,12–23^. The archetypal nano design is the so called Aharonov-Bohm ring, a nano constriction that can be coupled to quantum barriers, quantum dots, etc., that can give rise to asymmetrical line-shapes in the quantum conductance.

Within this context, bilayer graphene is a unique material system, not only because of its outstanding properties for technological applications^24–26^, but also because Fano resonances of different nature can arise in this material^27–31^. For instance, in gated bilayer graphene at room temperature light, electrons and phonons can confabulate to give rise to asymmetrical line shapes in the absorption spectra^27^. Here, it is quite relevant that a band gap be opened by electrical gating^25^ such that discrete phonons and continuous excitons be coupled. Fano phonon line shapes are also observed in the infrared spectra of bilayer graphene^28^. However, in this case the fundamental mechanism is the doping induced by gating rather than band gap opening. A radically different Fano resonance is also possible in bilayer graphene^29–31^. In fact, this resonance can arise without the need of an external continuum states source or an elaborated nano design^7,12–23,29–31^. In this case, it is necessary gapless bilayer graphene as well as low temperatures. The special dispersion relation of bilayer graphene^32,33^ makes that electrons have at the same time a propagating and discrete character^34,35^. Actually, the degree of coupling between confined and continuum states is ruled by the transversal component of the wave vector. By considering a potential barrier and adjusting the angle of incidence (the transversal wave vector) of the charge carriers on the barrier the propagating and discrete nature of electrons can converge to give rise to a Fano profile in the transmission properties^29–31^. This is unprecedented for a material and for graphene constitutes another exotic phenomenon like anti-Klein and Klein tunneling^34–37^, atomic collapse^38^, Hofstadter’s butterfly^39,40^ and negative refraction^41^ to mention a few. However, as far as we know this unusual and unprecedented phenomenon of Fano resonances in bilayer graphene has not been yet confirmed experimentally. This could obey the fact that: the asymmetrical line-shape of Fano resonances is manifested in the transmission probability or transmittance, for which a high degree of control of the angle of incidence of electrons is required^29–31^, issue that is not at all possible in current experiments; most of the exotic phenomena in graphene and related 2D materials have been demonstrated through transport measurements^36–41^; the lack of theoretical and experimental studies that address in a systematic way the impact of Fano resonances on the transport properties.

In this work, we address the unique and peculiar phenomenon of Fano resonances in bilayer graphene superlattices (BGSLs). The hybrid matrix method and the Landauer-Büttiker formalism have been used to obtain the transmission and transport properties. The evolution of the asymmetrical line-shape of Fano resonances in the transmission probability or transmittance is studied. In particular, the mentioned evolution is analyzed as a function of the superlattice period, the barriers height and the barriers and wells width. We pay special attention to the impact of Fano resonances on the transport properties. Our findings indicate that the intrinsic energy minibands of the superlattices structure can help to unequivocal identify the contribution of Fano resonances to the transport properties. Specifically, Fano resonances and minibands can couple in such a way that the linear-regime conductance presents signatures of this coupling, and hence of the presence of Fano resonances. Furthermore, by appropriately reducing the angular range for the computation of the transport properties it is possible to obtain conductance curves with line-shapes directly related to the Fano profile and its coupling with the miniband states. We hope that our results can help and encourage experimentalists to test this exotic phenomenon of Fano resonances in bilayer graphene.

## Metodology

**Figure 1:**
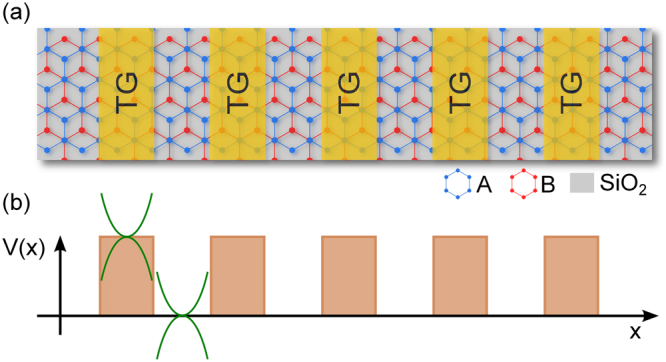
(**a**) Top view of the schematic representation of a bilayer graphene superlattice created by a periodic arrangement of top gate (TG) electrodes. Bilayer graphene, blue and red lattices, is typically placed on a non interacting substrate like SiO_2_ and a back gate not see from top view. (**b**) The resulting band-edge profile of (**a**), which is a periodic energy potential profile. The main effect of the electrostatic potential applied through TGs is a shifting of the Dirac paraboloids, paraboloids in the shaded area, proportional to the electrostatic field strength.

The system that we are interested in is a bilayer graphene superlattice. In particular, we will deal with BGSLs conceived by metallic electrodes arranged in periodic fashion, see Fig. 1a. Through the electrodes we can apply an electrostatic field perpendicularly to the graphene sheets in such way that the electrostatic potential in the two layers be the same. Keeping the same potential between the two layers ensures that the symmetry between them is preserved and consequently that no bandgap opening arises in the band structure. Then, the main effect of the electrostatic potential is a shifting of the Dirac paraboloids along the energy axis, see Fig. 1b. By arranging regions with and without electrostatic potential in periodic fashion we can generate a typical superlattice band-edge profile, see Fig. 1a. The transmission and transport properties of this system can be obtained through the hybrid matrix method^42,43^ and the Landauer-Büttiker formalism^44^, respectively. The basic information that we need in order to implement the mentioned methodologies is related to the wave functions and dispersion relations in the two regions that define the unit cell of the superlattice, that is, the region with and without electrostatic potential. So, we will present in first place the wave functions and dispersion relations to proceed with the generals of the hybrid matrix method and the Landauer-Büttiker formalism.

The wave functions and dispersion relation in the barrier region can be obtained by solving the following Dirac-like equation:

1 $$H\psi =E\psi ,$$

where the Hamiltonian is given as^32,33^,

2 $$H=-\frac{{\hslash }^{2}}{2m}(\begin{array}{cc}0 & {({q}_{x}-i{q}_{y})}^{2}\\ {({q}_{x}+i{q}_{y})}^{2} & 0\end{array})+V(x)(\begin{array}{cc}1 & 0\\ 0 & 1\end{array}),$$

here *q*_*x*_ and *q*_*y*_ are the quasiparticle wavevectors along the *x* and *y* directions respectively; *m* is the band effective mass with a value of 0.035 *m*_0_, being *m*_0_ the bare electron mass^29–31,45^; and *V*(*x*) = *V*_0_ represents the strength of the electrostatic potential.

The specific dispersion relation for this eigenvalue problem comes as,

3 $$E-{V}_{0}=\pm \frac{{\hslash }^{2}}{2m}{{\bf{q}}}^{2}\mathrm{.}$$

At this point it is important to mention that, in contrast to monolayer graphene, here in bilayer graphene there are four wave functions, two for propagating states and two more for evanescent-divergent states. The corresponding wave functions for propagating states can be written as

4 $${\psi }_{\pm }^{q}=(\begin{array}{c}1\\ {v}_{\pm }^{q}\end{array})\,{e}^{\pm i{q}_{x}x+i{q}_{y}y},$$

and the corresponding ones for evanescent-divergent states as

5 $${\psi }_{\pm }^{\beta }=(\begin{array}{c}1\\ {v}_{\pm }^{\beta }\end{array})\,{e}^{\pm {\beta }_{x}x+i{q}_{y}y},$$

with the coefficients ${v}_{\pm }^{q}$ and ${v}_{\pm }^{\beta }$ given as,

6 $${v}_{\pm }^{q}=-\frac{{\hslash }^{2}}{2m}\frac{{(\pm {q}_{x}+i{q}_{y})}^{2}}{E-{V}_{0}},$$

and

7 $${v}_{\pm }^{\beta }=-\frac{{\hslash }^{2}}{2m}\frac{{(\pm {\beta }_{x}+{q}_{y})}^{2}}{E-{V}_{0}}.$$

In these expressions *q*_*x*_ and *β*_*x*_ are:

8 $${q}_{x}=\sqrt{(\frac{2m}{{\hslash }^{2}})\,(E-{V}_{0})-{q}_{y}^{2}},$$

9 $${\beta }_{x}=\sqrt{(\frac{2m}{{\hslash }^{2}})\,(E-{V}_{0})+{q}_{y}^{2}}.$$

The general solution for the wave function can be written as a linear combination of the four eigenfunctions:

10 $$\psi (x,y)={A}_{+}{\psi }_{+}^{q}+{A}_{-}{\psi }_{-}^{q}+{B}_{+}{\psi }_{+}^{\beta }+{B}_{-}{\psi }_{-}^{\beta },$$

where *A*_+_, *A*_−_, *B*_+_, *B*_−_ is a set of expansion coefficients. Furthermore, for regions without electrostatic potential or better known as well regions the wavefunctions as well as the wave vectors can be obtained by simply setting *V*_0_ = 0.

With this information we can implement the standard transfer matrix method to compute the transmission properties. However, as we have shown^46–48^ this method has numerical instabilities and it is not suitable for the computation of the transmission properties of BGSLs. We have also shown that a better and reliable option is the hybrid matrix method^42,43,45^. The hybrid matrix method relies on writing the Dirac-like equation for bilayer graphene as an ordinary second order differential equation system of the Sturm-Liouville form^43^. Specifically, for bilayer graphene the matrix Sturm-Liouville equation comes as

11 $${\bf{L}}(x)\cdot {\bf{F}}(x)\equiv \frac{d{\bf{A}}(x)}{dx}+{\bf{Y}}(x)\cdot \frac{d{\bf{F}}(x)}{dx}+{\bf{W}}(x)\cdot {\bf{F}}(x)={{\bf{0}}}_{2\times 1}$$

where: A(*x*) = [**B**(*x*) · d**F** (*x*)/*dx* + **P**(*x*) · **F**(*x*)] is the so called associated-linear-form (ALF) of the operator **L**, with

12 $${\bf{B}}(x)=\frac{{\hslash }^{2}}{2m}[\begin{array}{cc}0 & 1\\ 1 & 0\end{array}];$$

13 $${\bf{P}}(x)={\bf{Y}}(x)=\frac{{\hslash }^{2}}{2m}{q}_{y}[\begin{array}{cc}0 & 1\\ -1 & 0\end{array}];$$

14 $${\bf{W}}(x)=[\begin{array}{cc}{V}_{0}-E & \frac{{\hslash }^{2}}{2m}{q}_{y}^{2}\\ \frac{{\hslash }^{2}}{2m}{q}_{y}^{2} & {V}_{0}-E\end{array}].$$

Within this context, the problem is reduced to find the linearly independent solutions

15 $${\bf{F}}(x)=|\begin{array}{c}{\psi }_{1}(x)\\ {\psi }_{2}(x)\end{array}|={{\bf{F}}}_{0}\,{e}^{ikx},$$

as well as the corresponding eigenvalues *k*. In fact, we can find them for the regions with and without electrostatic potential.

With the solutions **F**(*x*) and ALFs **A**(*x*) we can define the hybrid matrix. In fact, the hybrid matrix is the mathematical entity that relates the solutions and the ALFs between two regions, connected by a certain domain, in mixed fashion. In our case, we have a semi-infinite left region connected to a semi-infinite right region by a domain that is the superlattice structure. In mathematical terms we have

16 $$|\begin{array}{c}{\bf{F}}({\rm{L}}:{x}_{L})\\ {\bf{A}}({\rm{R}}:{x}_{R})\end{array}|=\,{\bf{H}}({x}_{R};{x}_{L})\cdot |\begin{array}{c}{\bf{A}}({\rm{L}}:{x}_{L})\\ {\bf{F}}({\rm{R}}:{x}_{R})\end{array}|.$$

Here, the hybrid matrix **H** is determined by the characteristics of the superlattice structure, specifically by the particularities of the solutions and ALFs in the barrier and well regions. Actually, we can define hybrid matrices for the well and barrier regions, and more importantly, irrespective of the particularities of these regions, the hybrid matrix can adopt the following general form

17 $$\begin{array}{ccc}{\bf{H}}(d) & = & [\begin{array}{cccc}{{\bf{F}}}_{10} & {{\bf{F}}}_{20} & {{\bf{F}}}_{30}\,{e}^{i{k}_{3}(-d)} & {{\bf{F}}}_{40}\,{e}^{i{k}_{4}(-d)}\\ {{\bf{A}}}_{10}\,{e}^{i{k}_{1}(d)} & {{\bf{A}}}_{20}\,{e}^{i{k}_{2}(d)} & {{\bf{A}}}_{30} & {{\bf{A}}}_{40}\end{array}]\\  &  & \cdot {[\begin{array}{cccc}{{\bf{A}}}_{10} & {{\bf{A}}}_{20} & {{\bf{A}}}_{30}{e}^{i{k}_{3}(-d)} & {{\bf{A}}}_{40}{e}^{i{k}_{4}(-d)}\\ {{\bf{F}}}_{10}{e}^{i{k}_{1}(d)} & {{\bf{F}}}_{20}{e}^{i{k}_{2}(d)} & {{\bf{F}}}_{30} & {{\bf{F}}}_{40}\end{array}]}^{-1},\end{array}$$

where *d* represents the width of the corresponding region. With the hybrid matrices of the well (**H**_*w*_) and barrier (**H**_*b*_) we can find the hybrid matrix of the unit cell of the superlattice (**H**_*uc*_) and with it the hybrid matrix of the entire superlattice **H**(*x*_*R*_;*x*_*L*_), for more details see ref.^45^.

In order to obtain the transmission probability or transmittance it is important to remember that the general solution is a linear combination of the four individual solutions. Specifically, the general solution and the general ALF for the left semi-infinite region can be written as

18 $${{\bf{F}}}_{L}(x)={a}_{1}(L)|\begin{array}{c}{\varphi }_{10w}\\ {\phi }_{10w}\end{array}|\,{e}^{i{k}_{1}(x-{x}_{l})}+{a}_{2}(L)|\begin{array}{c}{\varphi }_{20w}\\ {\phi }_{20w}\end{array}|\,{e}^{i{k}_{2}(x-{x}_{l})}+{a}_{3}(L)|\begin{array}{c}{\varphi }_{30w}\\ {\phi }_{30w}\end{array}|\,{e}^{i{k}_{3}(x-{x}_{l})},$$

19 $${{\bf{A}}}_{L}(x)={a}_{1}(L)|\begin{array}{c}{\alpha }_{10w}\\ {\beta }_{10w}\end{array}|\,{e}^{i{k}_{1}(x-{x}_{l})}+{a}_{2}(L)|\begin{array}{c}{\alpha }_{20w}\\ {\beta }_{20w}\end{array}|\,{e}^{i{k}_{2}(x-{x}_{l})}+{a}_{3}(L)|\begin{array}{c}{\alpha }_{30w}\\ {\beta }_{30w}\end{array}|\,{e}^{i{k}_{3}(x-{x}_{l})},$$

here we have set *a*_4_(*L*) = 0 to avoid divergence at *x* → −∞. In similar fashion for the right semi-infinite region,

20 $${{\bf{F}}}_{R}(x)\equiv {\psi }_{R}(x)={a}_{1}(R)|\begin{array}{c}{\varphi }_{10w}\\ {\phi }_{10w}\end{array}|\,{e}^{i{k}_{1}(x-{x}_{r})}+{a}_{4}(R)|\begin{array}{c}{\varphi }_{40w}\\ {\phi }_{40w}\end{array}|\,{e}^{i{k}_{4}(x-{x}_{r})},$$

21 $${{\bf{A}}}_{R}(x)={a}_{1}(R)|\begin{array}{c}{\alpha }_{10w}\\ {\beta }_{10w}\end{array}|{e}^{i{k}_{1}(x-{x}_{r})}+{a}_{4}(R)|\begin{array}{c}{\alpha }_{40w}\\ {\beta }_{40w}\end{array}|{e}^{i{k}_{4}(x-{x}_{r})},$$

where *a*_2_(R) = 0 and *a*_3_(R) = 0 because there is no reflected wave anymore and to avoid divergence at *x* → +∞, respectively.

With the help of these expressions it is possible to find relationships between the coefficients of the general solution of the left and right semi-infinite regions, namely:

22 $$|\begin{array}{c}{a}_{2}({\rm{L}})/{a}_{1}({\rm{L}})\\ {a}_{3}({\rm{L}})/{a}_{1}({\rm{L}})\\ {a}_{1}({\rm{R}})/{a}_{1}({\rm{L}})\\ {a}_{4}({\rm{R}})/{a}_{1}({\rm{L}})\end{array}|={[{{\bf{M}}}_{1}-{\bf{H}}({x}_{R},{x}_{L})\cdot {{\bf{M}}}_{2}]}^{-1}\cdot {\bf{H}}({x}_{R},{x}_{L})\cdot |\begin{array}{c}{{\bf{A}}}_{10}\\ {{\bf{0}}}_{2\times 1}\end{array}|-|\begin{array}{c}{{\bf{F}}}_{10}\\ {{\bf{0}}}_{2\times 1}\end{array}|\},$$

where:

23 $${{\bf{M}}}_{1}=(\begin{array}{cccc}{{\bf{F}}}_{20} & {{\bf{F}}}_{30} & {{\bf{0}}}_{2\times 1} & {{\bf{0}}}_{2\times 1}\\ {{\bf{0}}}_{2\times 1} & {{\bf{0}}}_{2\times 1} & {{\bf{A}}}_{10} & {{\bf{A}}}_{40}\end{array});$$

24 $${{\bf{M}}}_{2}=(\begin{array}{cccc}{{\bf{A}}}_{20} & {{\bf{A}}}_{30} & {{\bf{0}}}_{2\times 1} & {{\bf{0}}}_{2\times 1}\\ {{\bf{0}}}_{2\times 1} & {{\bf{0}}}_{2\times 1} & {{\bf{F}}}_{10} & {{\bf{F}}}_{40}\end{array}).$$

As the transmittance is given as $T={|\frac{{a}_{1}({\rm{R}})}{{a}_{1}({\rm{L}})}|}^{2}$, eq. (22), that depends directly of the hybrid matrix, is the fundamental equation to compute this fundamental physical quantity. Here, it is important to mention that the transmittance is in general a function of the energy and angle of incidence, *T* = *T*(*E*,*θ*).

With the transmittance at hand we can compute the transport properties readily by implementing the Landauer-Büttiker formalism^44^. In concrete, the linear-regime conductance can be obtained by summing over all transmission channels,

25 $$\frac{G}{{G}_{0}}={E}_{F}^{\ast }\,{\int }_{-\frac{\pi }{2}}^{\frac{\pi }{2}}\,T({E}_{F}^{\ast },\theta )\,\cos \,\theta \,d\theta ,$$

where ${E}_{F}^{\ast }=\frac{{E}_{F}}{{E}_{0}}$ is the dimensionless Fermi energy with *E*_0_ = *V*_0_, and ${G}_{0}=\frac{2{e}^{2}{L}_{y}{E}_{0}}{{h}^{2}{v}_{F}}$ the fundamental conductance factor. To calculate the linear-regime conductance for a specific angular range (*G* ^Δ*θ*^), we can reduce the integration limits in eq. (25), such that:

26 $$\frac{{G}^{{\rm{\Delta }}\theta }}{{G}_{0}}={E}_{F}^{\ast }\,{\int }_{-\theta }^{\theta }\,T({E}_{F}^{\ast },\theta )\,\cos \,\theta \,d\theta .$$

This expression will be useful in trying to know the particular shape of the conductance curves in the angular range at which Fano resonances dominate transport.

## Results and Discussion

### Bilayer graphene single barriers

As our main goal is to unveil the impact of Fano resonances on the transport properties we will proceed to analyse in the first place a single barriers (BGSBs), which is the most simple system, then we will study the case of double barriers (BGDBs) and finally a finite superlattice, with nine periods, will be addressed. Here, it is important to mention that even when the transmission properties of BGSBs and BGDBs are already reported^29–31^ we will present them in order to carry out a thorough evaluation and most importantly to determine in a precise way the energy regions in which Fano resonances contribute in a more significant way to the transport properties.

**Figure 2:**
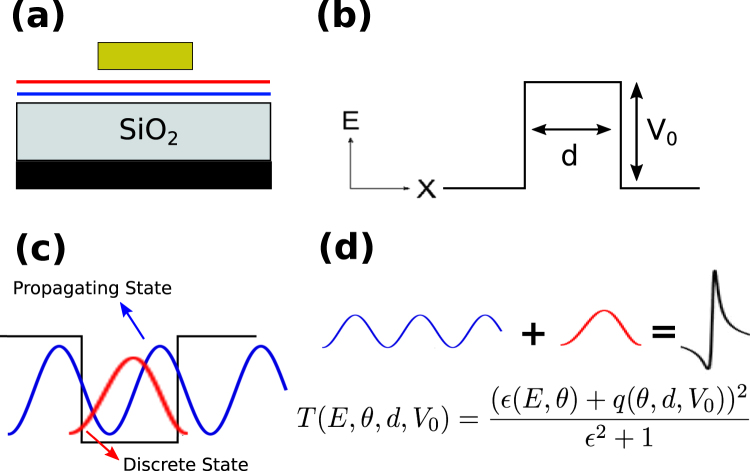
(**a**) Cross-section of the possible device for bilayer graphene single barriers. As in the case of BGSLs (Fig. 1a) graphene layers are placed between a top-gate, a SiO_2_ substrate and a back-gate in order to generate the (**b**) band-edge profile of a single barrier. (**c**) From the perspective of holes a single barrier in bilayer graphene actually represents a quantum well. Moreover, the quantum well can hold propagating and discrete states as a consequence of the number of channels within it. (**d**) Schematic representation of the output of the coupling of an extended state and a discrete one. In the case of bilayer graphene the transmission line-shape will depend on the energy, angle of incidence as well as barrier width and height.

As we can corroborate in the methodology there are four solutions or eigenfunctions for gapless bilayer graphene. So, in principle, by appropriately nanostructuring this material we can create the conditions to have in the same energy region propagating and discrete electron states, and hence the conditions to get Fano resonances. In Fig. 2 we show a schematic representation of the nanostructuring that we are dealing with. Graphene layers are placed on a non-interacting substrate, typically SiO_2_, top and back gates are incorporated to control the barrier characteristics as well as the Fermi energy of electrons, see Fig. 2a. The net effect of the electrostatic field induced by the top gate is a shift of the Dirac paraboloids in the energy axis. With this shifting a stepwise band-edge profile for the conduction band is generated, see Fig. 1b. Even though the region inside the barrier is not allowed for electrons, for holes it constitutes a resonant cavity, which in principle can give rise to propagating and discrete states, see Fig. 2c. By adjusting the angle of incidence, the barrier width and barrier height propagating and discrete states can converge to give place to Fano resonances in the transmission spectra, see Fig. 2d. The asymmetrical line-shape characteristic of Fano resonances will depend on the angle of incidence, the barrier width and barrier height, see Fig. 2d.

**Figure 3:**
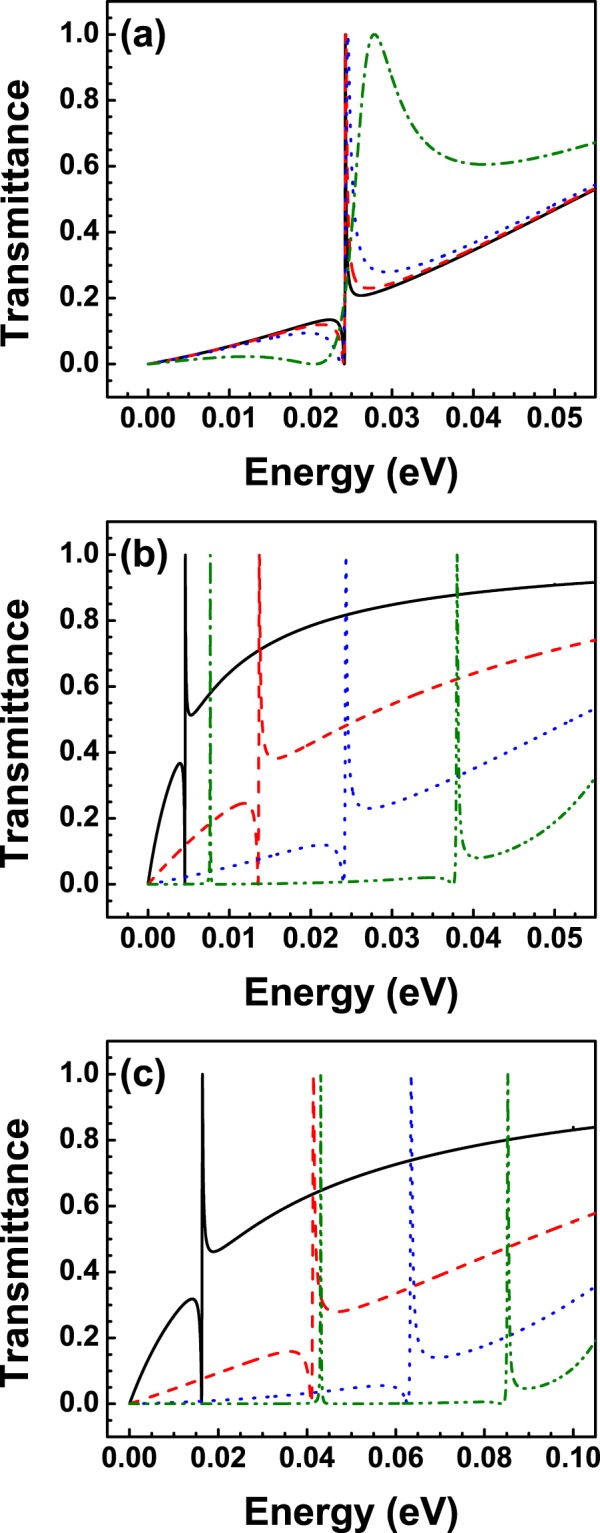
Transmittance as function of the energy for bilayer graphene single barriers. (**a**) Fano resonances for various angles of incidence: 1° (solid-black line), 3° (dashed-red line), 5° (dotted-blue line) and 15° (dash-dotted-green line). The width and height of the barrier remain fixed at 10 nm and 50 meV, respectively. (**b**) Evolution of Fano resonances for different barrier widths *dB*: 3 nm (solid-black line), 6 nm (dashed-red line), 10 nm (dotted-blue line) and 20 nm (dash-dotted-green line). The angle of incidence and the barrier height considered are 3° and 50 meV, respectively. (**c**) The same as in (**b**) but here *V*_0_ = 100 meV.

The results of the transmission probability or transmittance for BGSBs are presented in Fig. 3. The transmittance as a function of the energy for different angles of incidence is shown in Fig. 3a. The angles of incidence chosen are 1°, 3°, 5° and 15°, solid-black, dashed-red, dotted-blue and dash-dotted-green lines, respectively. The barrier width and barrier height considered are 10 nm and 50 meV, respectively. As we can notice the asymmetrical line-shape typical of Fano resonances arises in the transmission spectra. The asymmetrical line-shape is acute and well-defined for small angles and as the angle of incidence increases Fano resonance broadens and eventually for large angles the asymmetrical line-shape is lost. It is also important to remark that the energy location (*E* ≈ *V*_0_/2) of Fano resonances is practically unchanged as the angle of incidence grows. This feature is quite important because the contribution of Fano resonances to the transport properties will be located precisely at that specific energy. Then, in principle, we can track and discriminate the contribution of Fano resonances to the transport properties. In Fig. 3b we show how Fano resonances behave as the width of the barrier changes. We have kept fixed the angle of incidence and the height of the barrier at *θ* = 3° and *V*_0_ = 50 meV, respectively. As we can notice by changing the width of the barrier the Fano resonance is shifting to higher energies, that is, the resonance undergoes a blue shifting. The resonance that was originally located close to 5 meV for 3 nm is gradually shifting to: 15 meV for 6 nm, 25 meV for 10 nm and 40 meV for 20 nm. We can also see that the Fano profile deforms gradually and tends to become a Breit-Wigner resonance profile. Furthermore, a Breit-Wigner resonance arises in the low energy side of the transmission spectrum for the case of 20 nm. A similar shifting for the Fano resonance is obtained by increasing the height of the barrier, see Fig. 1c. In particular, by changing the barrier height from 50 meV to 100 meV the Fano resonance shifts nearly 10 meV, 25 meV, 37 meV and 45 meV for 3 nm, 6 nm, 10 nm and 20 nm, respectively. The Breit-Wigner resonance in the low energy side also blue shifts, approximately 35 meV. At this point, it is important to mention that the specific energies of the confined states can be computed by writing the eigenvalue problem of BGSBs in terms of the eigenbasis of *σ*_*x*_, *σ*_*x*_ *ψ*_±_ = ±*ψ*_±_, as well as by taking advantage that *ψ*_±_ are uncoupled at normal incidence^34^. In fact, *ψ*_−_ is the component that represents the confined states inside the barrier. The details of the method for the calculation of confined states are presented in the supplementary material. Likewise, the specific values of the confined states for the cases of BGSBs treated in Fig. 3 are computed and shown in the supplementary material. Despite confined states are calculated at normal incidence in some cases there is a good agreement with respect to energy location of the Fano resonances, see Table 1 for an explicit comparison. Here, it is also important to remark that once the normal incidence condition is relaxed the mixing between *ψ*_±_ gives rise to asymmetrical resonances in the transmission spectra. Actually, these resonances are pretty narrow near normal incidence because their width is proportional to ${q}_{y}^{2}$ ^34^. Furthermore, for narrow barriers, states with weaker confinement, it seems that the mixing is stronger to such extent that there is not at all correspondence between the energy of confined states and the energy location of Fano resonances.

**Table 1:** Comparison of the energies of the confined states obtained by the method presented in the supplementary material and the energies of the Fano resonances found in the transmission spectra of BGSBs.

	${\boldsymbol{\eta }}{\boldsymbol{=}}\frac{{\bf{3}}}{{\bf{50}}}$	${\boldsymbol{\eta }}{\boldsymbol{=}}\frac{{\bf{6}}}{{\bf{50}}}$	${\boldsymbol{\eta }}{\boldsymbol{=}}\frac{{\bf{10}}}{{\bf{50}}}$	${\boldsymbol{\eta }}{\boldsymbol{=}}\frac{{\bf{20}}}{{\bf{50}}}$	${\boldsymbol{\eta }}{\boldsymbol{=}}\frac{{\bf{3}}}{{\bf{100}}}$	${\boldsymbol{\eta }}{\boldsymbol{=}}\frac{{\bf{6}}}{{\bf{100}}}$	${\boldsymbol{\eta }}{\boldsymbol{=}}\frac{{\bf{10}}}{{\bf{100}}}$	${\boldsymbol{\eta }}{\boldsymbol{=}}\frac{{\bf{20}}}{{\bf{100}}}$
*E*_*b*1_	0.52	7	26	46	4	37	78	98
*E*_*FR*_	4	14	24	38	16	41	63	85

We have defined the ratio between the barrier width and barrier height *η* = *dB*/*V*_0_ as a parameter to characterize the barrier. The energies are given in meV and the barrier width in nm.

**Figure 4:**
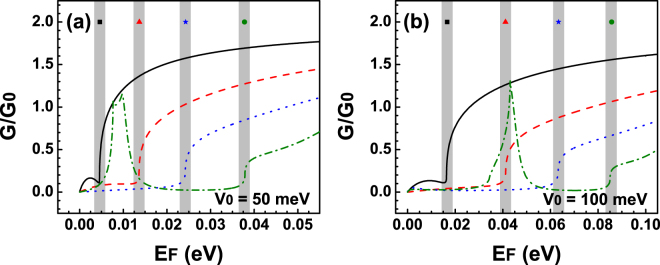
Conductance versus the Fermi energy *E*_*F*_ for bilayer graphene single barriers. (**a**) Conductance for various widths of the barrier *dB*: 3 nm (solid-black line), 6 nm (dashed-red line), 10 nm (dotted-blue line) and 20 nm (dash-dotted-green line). The barrier height in the cases is 50 meV. (**b**) The same as in (**a**) but here *V*_0_ = 100 meV. The shaded vertical stripes highlight the location of the Fano resonances.

**Figure 5:**
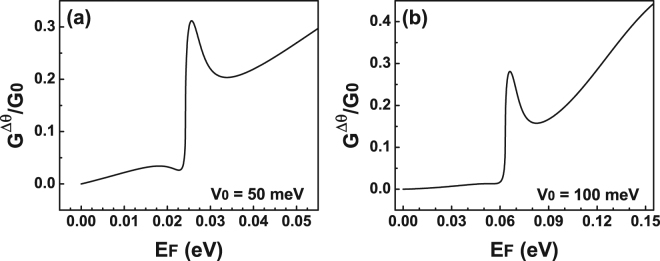
Conductance versus the Fermi energy for BGSBs in the angular range (−*π*/12, *π*/12) at which Fano resonances are preponderant. The heights of the barrier considered are (**a**) 50 meV and (**b**) 100 meV. The barrier width in both cases is 10 nm.

Now, having clear how Fano resonances change by the angle of incidence, the width and height of the barrier, we are in position of evaluate its impact on the transport properties. In Fig. 4 we show the linear-regime conductance as function of the Fermi energy for (a) *V*_0_ = 50 meV and (b) *V*_0_ = 100 meV. The barrier widths are the same as in the case of the transmittance, that is, the solid-black, dashed-red, dotted-blue and dash-dotted-green curves correspond to 3 nm, 6 nm, 10 nm and 20 nm, respectively. As we can see in both cases *V*_0_ = 50 meV and *V*_0_ = 100 meV the conductance behaves in the same way in general terms. For instance, in the case of the narrower barrier 3 nm, the conductance is practically zero up to 5 meV (15 meV) for *V*_0_ = 50 meV (*V*_0_ = 100 meV). At that specific energy the conductance presents a sudden rise that coincides quite well with the energy location of the Fano resonances. In order to highlight the energy location of Fano resonances we have included shaded vertical stripes. Furthermore, the shape of the conductance curves remains the same no matter the width of the barrier. The main change that we can notice is that the sudden increase is taking place at higher energies. Those energies are precisely the energies at which Fano resonances are presented in the transmission spectra. In the case of 20 nm we can also see a peak that is directly related to the Breit-Wigner resonances that arise in the transmittance, see the dash-dotted-green curves in Fig. 3. Despite the simplicity of a single barrier we were able to identify the energy region at which Fano resonances contribute to the transport properties. Even more important, this region could serve as hallmark of the existence of this rather exotic phenomenon of Fano resonances in bilayer graphene as well as help our experimental counterparts to prove it. At this point, it is also important to mention that the advance and refinement of the experimental techniques allow nowadays to discriminate the angular contribution of Dirac electrons to the transport properties in single-barrier graphene devices^49–52^. In particular, by tilting the top-gate electrodes it was possible to determine the mentioned contribution. This is quite appealing for us because we can compute the conductance in the angular range at which Fano resonances are preponderant. In fact, in Fig. 5 we show the results for the conductance as a function of the Fermi energy in the angular range (−*π*/12, *π*/12) at which the Fano resonances are relevant. Figure 5a,b correspond to *V*_0_ = 50 meV and *V*_0_ = 100 meV, respectively. The width of the barrier considered in both cases is *dB* = 10 nm. By reducing the angular range we can see that an asymmetrical lines-shape also arises for the conductance at precisely the energies at which the Fano resonances are manifested in the transmission spectra. These Fano profiles in the conductance are undoubtedly attributed to Fano resonances in the transmittance curves. Then, it is possible that with angular transport measurements the existence of Fano resonances can be proven.

### Bilayer graphene double barriers

**Figure 6:**
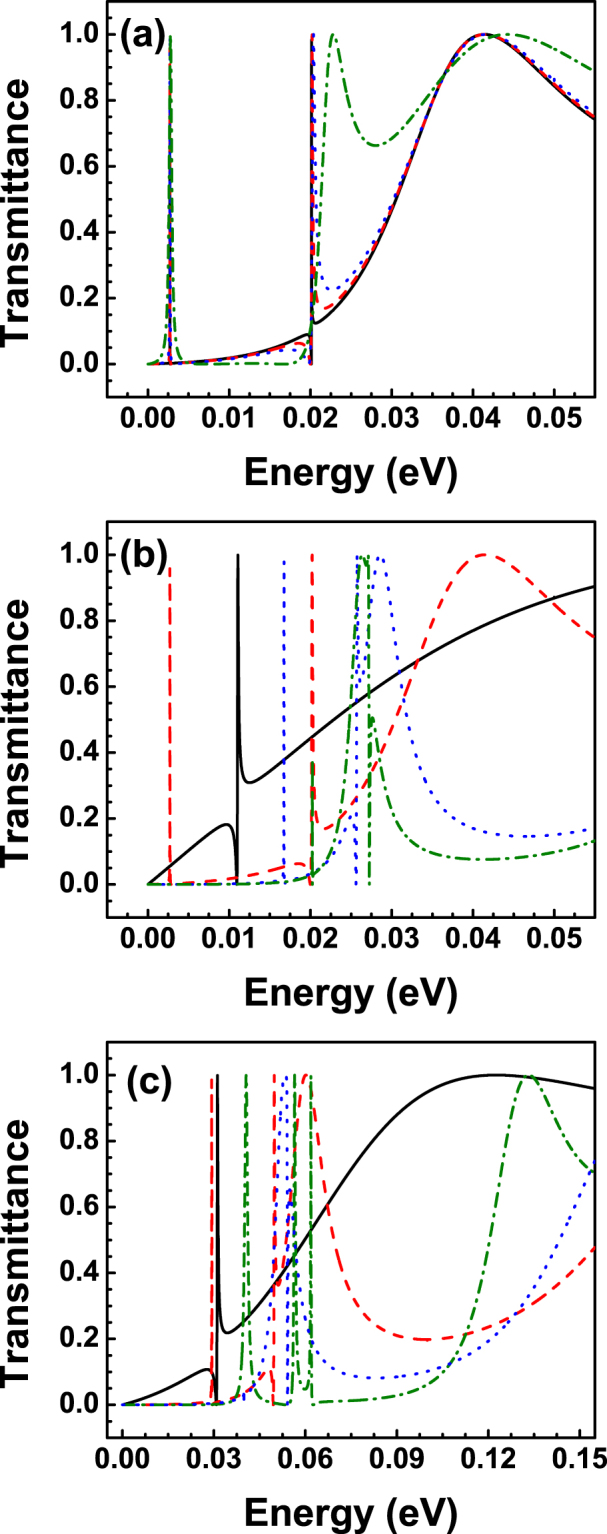
Transmittance as function of the energy for BGDBs. (**a**) Transmission spectra for different angles of incidence: 1° (solid-black line), 3° (dashed-red line), 5° (dotted-blue line) and 15° (dash-dotted-green line). The widths of barriers-well and the heights of the barriers considered were *dB* = *dW* = 6 nm and *V*_0_ = 50 meV, respectively. (**b**) Evolution of the transmittance for different widths of barriers-well *dB* = *dW* $=:$ 3 nm (solid-black line), 6 nm (dashed-red line), 9 nm (dotted-blue line) and 10 nm (dash-dotted-green line). The angle of incidence and the height of the barriers in this case are *θ* = 3° and *V*_0_ = 50 meV, respectively. (**c**) Similar to (**b**) but here the widths considered are 3 nm (solid-black line), 6 nm (dashed-red line), 7 nm (dotted-blue line) and 9 nm (dash-dotted-green line). In this case the height of the barriers has been increased to *V*_0_ = 100 meV.

**Figure 7:**
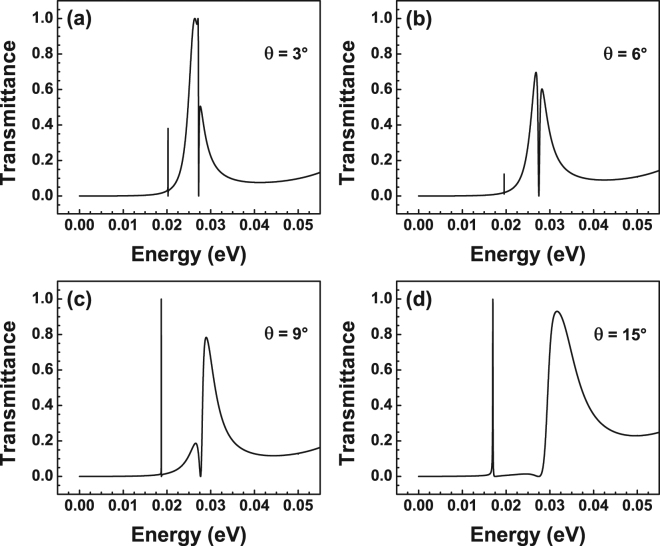
Evolution of hybrid Fano resonances for different angles of incidence: (**a**) 3°, (**b**) 6°, (**c**) 9° and (**d**) 15°. The width and height of the barriers are 10 nm and 50 meV, respectively. As in the case of Fano resonances the hybrid line-shape is: well defined at small angles; deformed and eventually lost as the angle increases.

Now, it is turn to analyse the case of BGDBs. Here, it is important to stress out that this case is more versatile because the well region can incorporate its own propagating and/or discrete states that will enrich the features of the transmission spectra. Our results for this specific case are shown in Fig. 6. The evolution of the transmittance for various angles of incidence is presented in Fig. 6a. The height of the barriers and the widths of barrier and well are *V*_0_ = 50 meV and *dB* = *dW* = 10 nm. Here, we are considering *dB*_1_ = *dB*_2_ = *dB* as well as *V*_*B*1_ = *V*_*B*2_ = *V*_0_, that is, we are dealing with symmetric barriers. As we can notice Fano resonances arise at approximately 20 meV, which represents a small red-shift with respect to BGSBs. We also found that the asymmetrical line-shape of the resonances is preserved only at small angles. Two additional features have arisen as a result of the well region: (1) In the low energy side there is a Breit-Wigner resonance at about 2.5 meV, which remains at the same energy irrespective of the angle of incidence; (2) In the high energy side we can see an extended resonance at 40 meV, which tends to broaden as the angle of incidence increases, see the dash-dotted-green curve. So, in principle, the interplay of these resonances with the asymmetrical line-shape associated to the barrier regions can give rise to new resonance characteristics. These new characteristics can change the conductance landscape and possibly can give place to special features in the transport properties that can serve as hallmarks of the existence of Fano resonances. In fact, in Fig. 6b we can see that these new characteristics arise by adjusting the widths of the barriers and the well, while remaining the angle of incidence and the height of the barriers at constant values, 3° and 50 meV, respectively. For instance, in the case of *dB* = *dW* = 3 nm we can see a transmission spectrum typical of BGSBs with a Fano resonance around 10 meV. In this case the well is so narrow that its characteristics are not at all manifested in the transmission spectrum. By increasing the widths to *dB* = *dW* = 6 nm resonances in the low and high energy side of the spectrum come into play as in Fig. 6a. The Fano resonance also blue-shifts roughly 10 meV with respect to the case of 3 nm. As far as we have corroborated Fano resonances are a characteristic that come from the barrier region and the low and high energy side resonances come mainly from the well. So, a simultaneous increase of the width of the barriers and the well makes that the resonances approach each other and eventually that the interplay between them gives rise to new resonance profiles. Actually, this is the case for 9 nm and 10 nm, respectively. Specifically, for 9 nm the Fano resonance and the resonance at the high energy side are pretty close, but not enough to change their own profiles. In the case of 10 nm the resonances have merged to give rise to a new resonance profile, at about 27 meV, that resembles to what it is known in optics as hybrid Fano resonance^53^. Similar transmission spectra are obtained for *V*_0_ = 100 meV. The main changes with respect to 50 meV are the blue-shifting of the overall spectra as well as that the hybrid Fano resonance arises at narrower widths, see the case of 7 nm. We also notice that the hybrid Fano resonance eventually splits into two weakly coupled Breit-Wigner resonances, see the dash-dotted-green curve that corresponds to 9 nm. Here, it is worth mentioning that hybrid resonances are well defined at small angles of incidence and that they tend to deform and eventually lose their hybrid line-shape as the angle grows, see Fig. 7. As in the case of BGSBs this opens the possibility to study the transport properties in the angular range in which hybrid resonances are preponderant as well as the opportunity to test the existence of these resonances by angular transport measurements.

**Figure 8:**
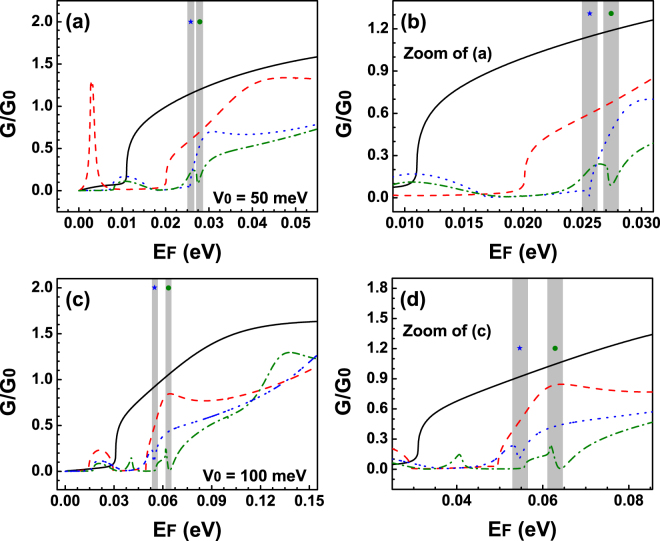
Conductance versus the Fermi energy *E*_*F*_ for BGDBs. (**a**) Conductance for different barriers-well widths. The widths and heights correspond to those used in Fig. 4b. (**c**) Similar to (**a**) but the parameters used are the ones that correspond to Fig. 4c. (**b**,**d**) Represent zooms of (**a**,**c**). These figures have the intention of magnify the region in which hybrid resonances are preponderant and even more important how these resonances defined the line-shape of the conductance curves. The shaded vertical stripes highlight the location of the Fano and the hybrid Fano resonances.

**Figure 9:**
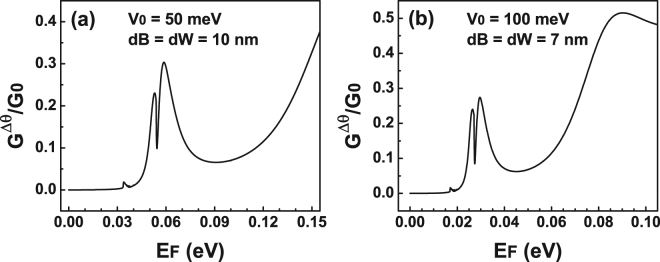
Conductance versus the Fermi energy for BGDBs in the angular range (−*π*/12, *π*/12) at which hybrid Fano resonances are preponderant. The heights (widths) of the barrier considered are (**a**) 50 meV (10 nm) and (**b**) 100 meV (7 nm).

Now, it is turn to analyse the transport properties of BGDBs. In Fig. 8 we show the linear-regime conductance versus the Fermi energy for (a) *V*_0_ = 50 meV and (b) *V*_0_ = 100 meV. The widths of the barriers and the well for Fig. 8a,b are the same as in Fig. 6b,c. As we can see for small widths the conductance is practically the same as for BGSBs, see the solid-black and dashed-red curves. For the case of 10 nm and 7 nm, dash-dotted-green curve in Fig. 8a and dotted-blue curve in Fig. 8b, the conductance presents some features that contrast with the other cases such is the case of the peak and minimum at around 27 meV and 55 meV respectively. These characteristics are shaped mostly by hybrid Fano resonances because their energy location coincides perfectly with the location of the hybrid line-shapes in the transmittance. We also see that in the case of 9 nm, dash-dotted-green curve in Fig. 8b, the peak-minimum region is steeper as well as its localization agrees quite well with the weakly coupled Breit-Wigner resonances of the transmission spectra, dash-dotted-green curve in Fig. 6c. So, these peak-minimum characteristics are directly related to the hybrid Fano resonances and in principle they can serve as a hallmark of the existence of these special resonances and consequently of the existence of the Fano resonances. Furthermore, by reducing the angular range for the conductance to (−*π*/12, *π*/12) we obtain that the conductance also exhibits the hybrid line-shape, see Fig. 9. This range is precisely the angular region at which the hybrid resonances are preponderant, Fig. 7. We can also notice that this region is totally identifiable and in principle can be detectable via angular transport measurements.

### Bilayer graphene superlattices

**Figure 10:**
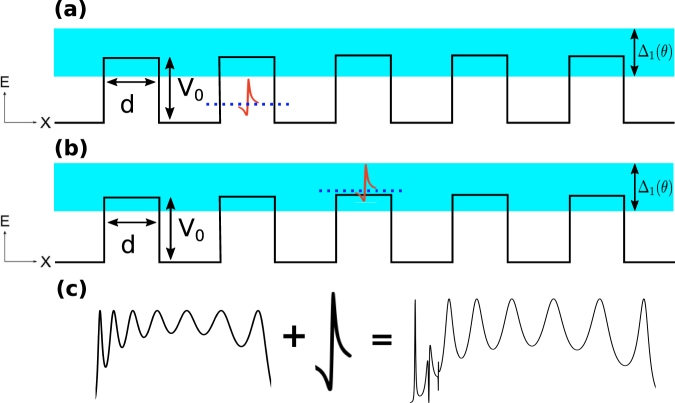
Schematic representation of the possible scenarios between Fano resonances and miniband states. (**a**) The Fano resonance (dotted-blue line) and the miniband (shaded cyan region) lie in different energy regions such that there is no coupling between them. (**b**) The Fano resonance and the miniband lie in the same energy region and their coupling can take place. (**c**) Coupling of a Fano resonance and miniband states giving rise to new features in the transmission spectrum.

**Figure 11:**
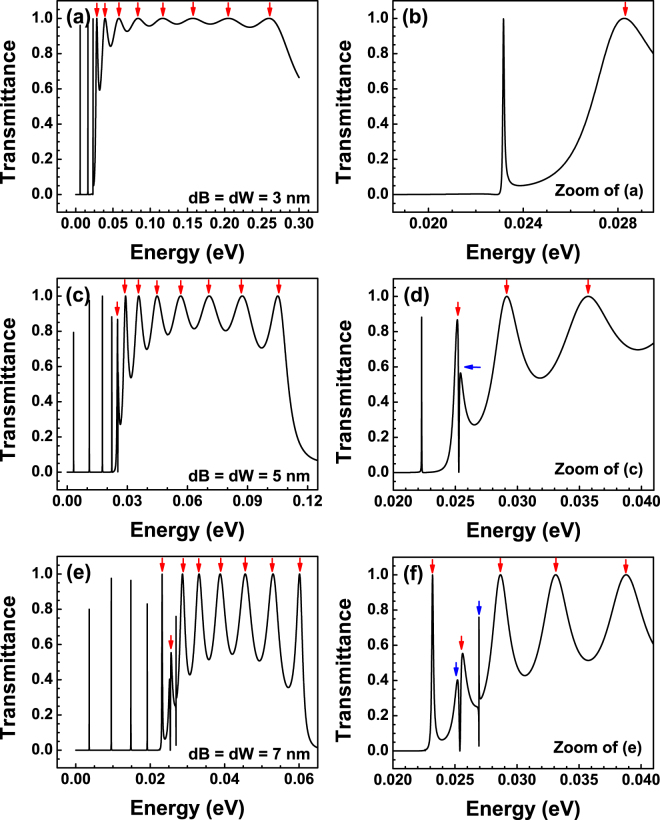
Transmittance of BGSLs for different barriers-wells widths *dB* = *dW*: (**a**) 3 nm, (**c**) 5 nm and (**e**) 7 nm. In all these cases the height of the barriers, the angle of incidence and the superlattice period were set at 50 meV, 3° and 9, respectively. In order to have a better view of the Fano resonance and its coupling with miniband states zooms of the considered transmission spectra are shown. Specifically, (**b**,**d**,**f**) represent zooms of (**a**,**c**,**e**), respectively.

**Figure 12:**
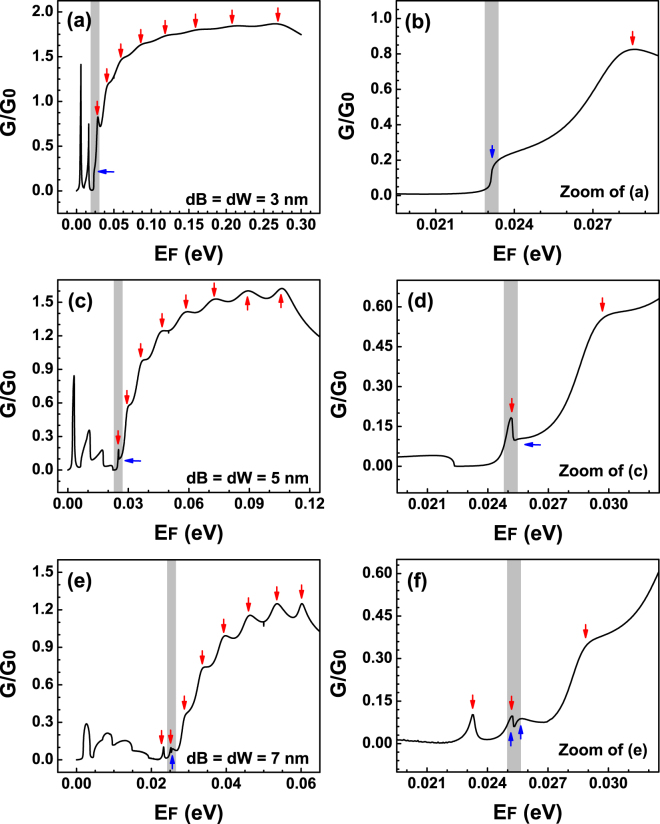
Conductance of BGSLs for different barriers-wells widths *dB* = *dW*: (**a**) 3 nm, (**c**) 5 nm and (**e**) 7 nm. In order to have a better perspective of the hallmark on the conductance of the contribution of Fano resonances and its coupling with miniband states the energy range has been reduced to the energy region at which the coupling is preponderant. The other structural parameters of the superlattice are the same as in Fig. 11. The shaded vertical stripes highlight the energy region at which the coupling is taking place.

**Figure 13:**
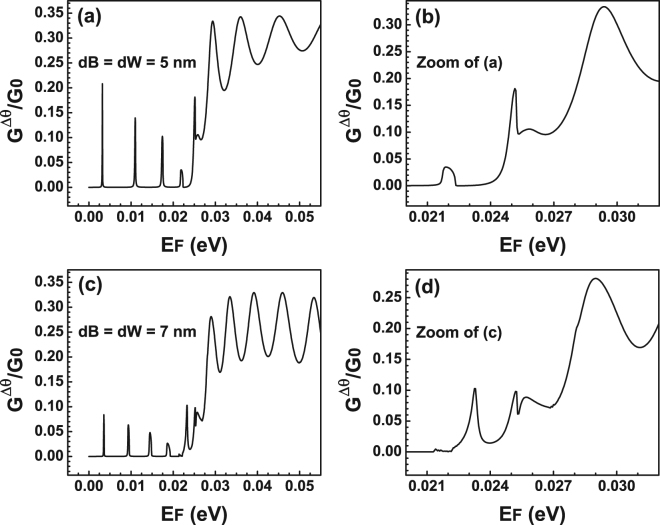
Conductance of BGSLs for the angular range at which the coupling between Fano resonances and miniband states is dominant. In this case the angular range has been reduced with respect to the cases of single and double barriers. In particular, the angular range considered is (−*π*/18, *π*/18). (**a**,**c**) Correspond to the widths of Fig. 12c,e, respectively. The other structural parameters of the superlattice are the same as in the preceding figures, Figs 11 and 12. (**b**,**d**) Represent zooms of (**a**,**c**), respectively.

Now it is time to analyse the more general case of bilayer graphene superlattices. Here, it is worth mentioning that one of the most remarkable characteristics of practically any superlattice is the formation of the so-called minibands. In this regard the periodic arrangement of barriers and wells in bilayer graphene is not the exception. The most noteworthy difference of BGSLs with respect to superlattices of conventional materials is that minibands depend strongly on the angle of incidence in the former case. As we have corroborated in the case of single and double barriers Fano resonances represent an intrinsic characteristic of bilayer graphene. Moreover, these special resonances also depend strongly on the angle of incidence. Then, at first instance, these characteristics, minibands and Fano resonances, can be located in different energy regions, however, at second instance, by appropriately adjusting the fundamental parameters of the superlattice such as the angle of incidence, the widths of barriers and wells, the heights of barriers and the superlattice period it is possible to tune the minibands and Fano resonances at the same energy region. In Fig. 10a,b we show the schematic representation of these possibilities. More importantly the interplay of minibands and Fano resonances can give rise to new features in the transmission spectra. The possible resulting line-shape of the mentioned interplay is shown schematically in Fig. 10c. In principle, these new features can be more intricate than the ones found in the case of double barriers, and in general they will depend on the location of the Fano resonance within the miniband. Our specific findings for a finite superlattice of nine periods are shown in Fig. 11. Three different widths of barriers-wells *dB* = *dW* have been considered: (a) 3 nm, (b) 5 nm and (c) 7 nm. The height of the barriers and the angle of incidence have remained fixed at 50 meV and 3°, respectively. For 3 nm we can see that the Fano resonance and the miniband lie in different energy regions consequently there is no coupling as well as no new features in the transmission spectrum. The Fano resonance is located at 23 meV, while the miniband starts at 28 meV and it has an energy width of 222 meV. By increasing *dB* = *dW* to 5 nm the Fano resonance and the miniband coincide in the same energy region, specifically the Fano resonance lies at the onset of the miniband. In this case the resulting line-shape corresponds to the type of hybrid Fano resonances. It is also important to note that the miniband width is reduced around 132 meV. Likewise, the Fano resonance and the miniband blue and red shift with respect to the case of 3 nm. A further increase of the barriers-wells width to 7 nm turns out in an additional reduction of the miniband width of nearly 53 meV as well as extra blue and red shifts of the Fano resonance and the miniband. The net result of these shifts and the miniband narrowing is that the Fano resonance blue shifts with respect to the onset of the miniband, in other words, the Fano resonance tends to be localized at higher energies within the miniband. In fact, by appropriately adjusting the fundamental parameters of the superlattice we can tune the position of the Fano resonance at practically any energy within the miniband. The coupling of the Fano resonance with the states along the miniband gives rise to new and unique features in the transmission spectra. Even more important, we were able to identify and discriminate the contribution of these new transmission line-shapes onto the transport properties. For instance, when the Fano resonance and the miniband are decoupled, Fig. 12a, the linear-regime conductance shows the typical sudden jump related to Fano resonances, in this case at 23 meV, as well as a broad peak with small oscillations that constitutes the contribution of the miniband. The red vertical arrows indicate that the small oscillations (peaks) are associated to states within the miniband. We can also see acute peaks in the low energy side which are associated to Breit-Wigner resonances. When the Fano resonance and the miniband are coupled at the onset of the latter, Fig. 12b, the conductance presents a distinctive feature at the onset of the broad peak that come from the miniband, see the blue vertical arrow. In fact, this distinctive feature is a small peak with a line-shape that contrasts with the typical line-shapes associated to the states of the miniband. A further penetration of the Fano resonance into the miniband, Fig. 12d, gives rise to a peculiar line-shape in the conductance. Specifically, notice the small notch, highlighted by blue arrows and the shaded vertical stripe, at about 25 meV. It is worth mentioning that the location of this notch agrees well with the localization of the new features of the transmission spectrum of Fig. 11c. Here, it is also important to remark that despite transport is practically dominated by the miniband and the Breit-Wigner resonances it is still possible to identify and characterized the contribution of the new spectral features. Moreover, by reducing the angular range for the conductance it is possible to discriminate in a more transparent and direct way the contribution of the coupling of the Fano resonance and the states of the miniband. In Fig. 13 we show our results for the angular reduction of the conductance curves presented in Fig. 12d,f. In this case the angular range was reduced to (−*π*/18, *π*/18). As in the case of single and double barriers, the angular reduction modifies greatly the conductance curves to such an extent that the new conductance curves resemble in great extent to the corresponding curves of the transmission spectra.

### Discussion and important remarks

Finally, we want to discuss some important aspects about Fano resonances in bilayer graphene:

1. The first aspect that we want to address is the Fano-resonance profile. In fact, there is a formalism that provides a universal formula for the Fano-resonance profile in the context of coherent quantum transport^23^. This formalism solves important problems such as the nature of the parameter *q* and the width of the resonance. Taking into account that this formalism is based on the Green’s function method for quantum transport and that the Green function and the scattering formalism of quantum transport are equivalent^44^, in principle the universal formalism can be applied to our problem. In particular, it is quite interesting and relevant how the universal formula for the Fano-resonance profile will depend on the angle of incidence, since as we have corroborated throughout our study, the angle of incidence represents a preponderant parameter that determines in great extent the Fano-resonance profile. Considering the magnitude and relevance of this aspect, a thorough analysis, that go beyond the objectives of the present work, is required.
2. The second aspect that it is important to discuss is the one related to non-idealities such as substrate effects, impurity scattering and temperature disorder. In fact, these effects can can modify the fundamental characteristic of the band structure of bilayer graphene as well as destroy coherent quantum transport, and consequently jeopardizing the particular conditions that give rise to Fano resonances. For instance, substrates that interact strongly with bilayer graphene open a band gap and modify the dispersion relation^54^. On the other hand, if disorder, caused by impurity scattering or temperature, is relevant the transport will be predominantly diffusive rather than ballistic. Therefore, compromising the specific conditions to obtain Fano resonances. Fortunately, high-quality bilayer graphene samples, non-interacting substrates like SiO2 and very low temperatures can guarantee the conditions to obtain a gapless parabolic dispersion relation and coherent quantum transport in bilayer graphene. In fact, unconventional quantum Hall effect and 2*π* Berry phase associated to the gapless parabolic dispersion relation in bilayer graphene have been demonstrated experimentally^24^. In addition, it is well known that high quality of graphene samples ensures coherent quantum transport up to 80 K^37^. So, in principle, the detection of Fano resonances is achievable via low-temperature transport measurements.
3. The last aspect that we consider it is important to address is the one associated to relevant effects such as the bandgap opening, non parabolicity and warping. For instance, in order to preserve a gapless band structure in bilayer graphene the sheets need to be maintained at the same potential energy. Despite the sophistication of the experimental techniques there will be unavoidable differences between the potential energies of the graphene sheets, causing a bandgap opening in the band structure of bilayer graphene. This bandgap opening can modify significantly the fundamental properties of bilayer graphene. In fact, in the case of anti-Klein tunneling it has been reported that the bandgap opening can destroy it^55^. Regarding Fano resonances our results in BGSBs indicate that the asymmetrical line shape can be deformed and for a certain band gap practically destroyed, see the supplementary material for more details. In addition, band gap opening can activate phonons^27^ that in principle could be coupled with propagating or discrete electron states and/or could represent an additional scattering mechanism. As far as we know these phonons were observed at room temperature^27^, but if they are present at temperatures below liquid helium could affect in great extent the asymmetrical line shape associated to confined and continuum electron states. Non parabolicity and warping are effects that can also modify the transmission and transport properties in bilayer graphene structures. In specific, non parabolicity is relevant at energies of 390 meV. Then, if we want to limit this effect it is crucial to work in a reduced energy range, otherwise it will be relevant to know the possible changes that non parabolicity can caused to the transmission and transport properties. Up to this moment, the numerical degradation related to the transfer matrix approach and the Hamiltonian that describe band gap opening and non parabolicity impede us to see to what extent the Fano profile is modified by non parabolicity. The mentioned numerical degradation is presented in the corresponding section of the supplementary material. Lastly, warping in principle it is less relevant than the bandgap opening and non parabolicity^32^, however, effects such as temperature can induced natural distortions on the graphene sheets^56^. So, it is also interesting to know the impact of warping on the transmission and transport properties. A full analysis of band gap opening, non parabolicity and warping is needed and its possible consequences will be published elsewhere.

## Conclusions

In summary, we have addressed the exotic phenomenon of Fano resonances in bilayer graphene superlattices. The hybrid matrix method and the Landauer-Büttiker were implemented to unveil the Fano characteristics on the transmission and transport properties. Particularly, we find an asymmetrical line-shape, Fano profile, on the transmittance characteristics. The Fano profile is pretty sensitive to the electron angle of incidence, being well defined for small angles, and deformed and practically lost for large ones. We also find that the Fano resonances can be coupled with the energy minibands of the superlattice, giving rise to special transmission characteristics. Even more important, the Fano resonance by itself as well as its coupling with miniband states have direct and identifiable consequences on the transport properties. Specifically, well-defined conductance characteristics arise at Fermi energies at which the Fano resonance and its coupling are taking place. Furthermore, angular transport calculations provide conductance curves with practically the same line-shape of Fano resonances as well as the same profile of its coupling with miniband states. Then, we have shown that bilayer graphene superlattices are systems that can be helpful to obtain unequivocal characteristics in the transport properties related to existence of Fano resonances. We hope that our findings encourage further studies about this rather exotic phenomenon of Fano resonances in bilayer graphene, and furthermore, that our results help and encourage experimentalists to test this peculiar phenomenon.

## Electronic supplementary material

Supplementary Information
