# Supplementary material for: Fano resonances in bilayer graphene superlattices
Source: Sci Rep. 2017 Dec 1;7:16708. doi: 10.1038/s41598-017-16838-9 (PMC5711848; doi:10.1038/s41598-017-16838-9)
Supplement: Supplementary file 1 — Supplementary Information [file 41598_2017_16838_MOESM1_ESM.pdf]

Supplementary Information

Fano resonances in bilayer graphene superlattices

J. A. Briones-Torres and I. Rodríguez-Vargas

Unidad Académica de Física, Universidad Autónoma de Zacatecas, Calzada Solidaridad Esquina Con Paseo La Bufa S/N, 98060 Zacatecas, Zac., México.

S.I. Confined states in a rectangular barrier in gapless bilayer graphene

Chirality mismatch at normal incidence in bilayer graphene single barriers gives rise to the so-called cloaked states.[1] These states are of confined character and are practically invisible to states outside the barrier. In fact, it is possible to compute them by writing the eigenvalue problem in the basis of σ_x, $\sigma_x \psi_\pm = \pm\psi_\pm$,

$$\left(-\frac{\hbar^2}{2m}\frac{d^2}{dx^2} - q_y^2 \pm (V(x) - E)\right)\psi_\pm = 2q_y \frac{d\psi_\mp}{dx}. \tag{S.1}$$

At normal incidence $q_y = 0$ there is no mixing between the components $\psi_\pm$, then eq. (S.1) takes the form

$$\left(-\frac{\hbar^2}{2m}\frac{d^2}{dx^2} - q_y^2 \pm (V(x) - E)\right)\psi_\pm = 0. \tag{S.2}$$

The ψ_- component gives us the confined states, while the ψ_+ component represents the propagating states outside the barrier. In the case of rectangular barriers, $V(x) = V_0$ for $0 < x < dB$ and $V(x) = 0$ otherwise, the eigenfuction solutions are given as

$$\psi_-(x) = \begin{cases} C_L e^{\kappa_x x}, & x < 0, \\ A e^{iq_x x} + B e^{-iq_x x}, & 0 < x < dB, \\ C_R e^{-\kappa_x x}, & x > dB, \end{cases} \tag{S.3}$$

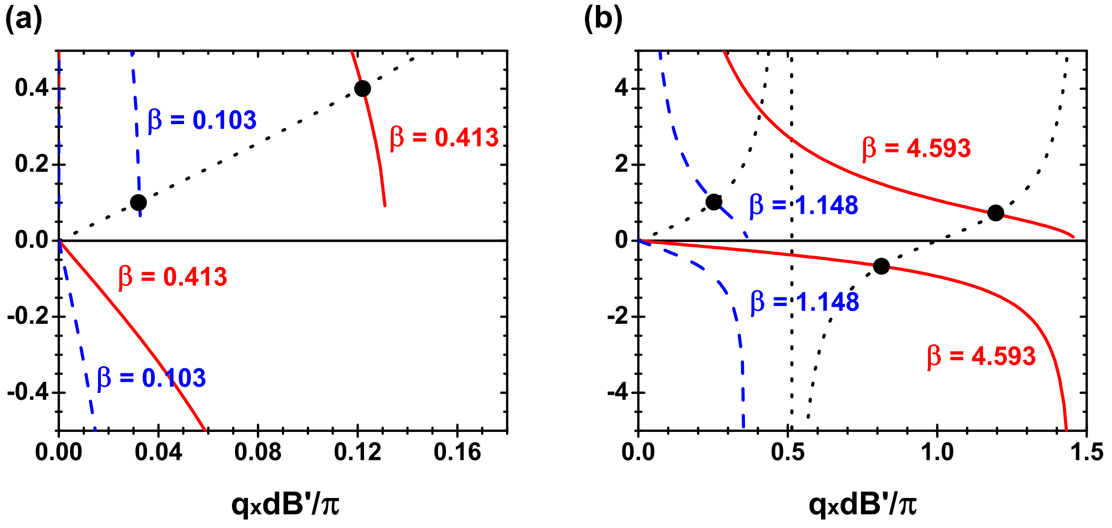

Fig. S1: Graphical solutions of eqs. (S.5) and (S.6) for confined states in bilayer graphene single barriers. The dotted-black lines represent $\tan(q_x dB')$, while the solid-red and dashed-blue curves correspond to the branches $\sqrt{\beta^2 - (q_x dB')^2}/q_x dB'$ and $-q_x dB'/\sqrt{\beta^2 - (q_x dB')^2}$. In all cases the height of the barrier is $V_0 = 50$ meV. In (a) the dashed-blue and solid-red curves correspond to $dB = 3$ nm ($\beta = 0.103$) and $dB = 6$ nm ($\beta = 0.413$) respectively, while in (b) the same type curves correspond to $dB = 10$ nm ($\beta = 1.148$) and $dB = 20$ nm ($\beta = 4.593$). The dotted-black vertical lines are the asymptotes of $\tan(q_x dB')$. The confined states are determined by the intersections of the dotted-black curves with the dashed-blue and solid-red ones.

where $q_x = \sqrt{2m(V_0 - E)/\hbar^2}$ and $\kappa_x = \sqrt{2mE/\hbar^2}$. The continuity condition of the wave function and its derivative at the boundaries, $x = 0$ and $x = dB$, allows us to obtain the transcendental equation that determine the bound states,[2]

$$\cot(q_x dB) = -\frac{1}{2}\left[\frac{\kappa_x}{q_x} - \frac{q_x}{\kappa_x}\right]. \tag{S.4}$$

Actually, this equation has two branches,

$$\tan(q_x dB') = \frac{1}{q_x dB'}\sqrt{\beta^2 - (q_x dB')^2}, \tag{S.5}$$

$$\tan(q_x dB') = -\frac{q_x dB'}{\sqrt{\beta^2 - (q_x dB')^2}}, \tag{S.6}$$

where $dB' = dB/2$ and $\beta^2 = 2mV_0 dB'^2/\hbar^2$. By solving these equations graphically we can obtain confined states by substituting the specific values of $q_x dB'$ in the following equation,

$$E_b = V_0 \left[1 - \frac{(q_x dB')^2}{\beta^2} \right]. \tag{S.7}$$

The graphical results for our cases of single barriers are presented in Figures S1 and S2.

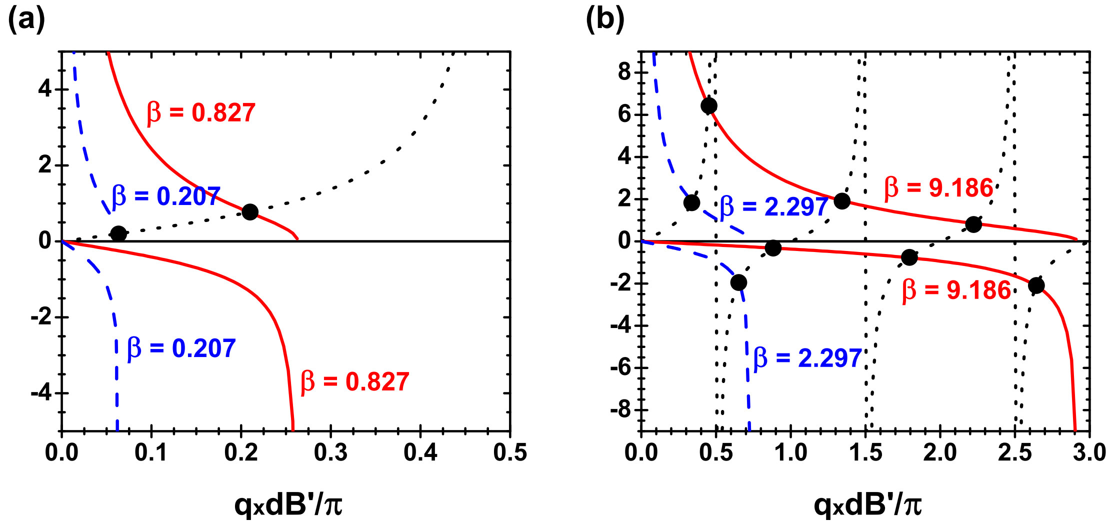

Fig. S2: The same as in Fig. S1 but here $V_0 = 100$ meV.

In Table S1 we show the specific energies of the confined states for the different cases of single barriers studied in the present work.

Table S1: Energies of the confined states for BGSBs computed with the values of $q_x dB'$ obtained with the graphical method, Figs. S1 and S2. The energies are given in meV, while the lengths in nm. β and $q_x dB'$ are dimensionless parameters.

β	V_0	dB'	$(q_x dB'/\pi)_{b1}$	E_{b1}	$(q_x dB'/\pi)_{b2}$	E_{b2}	$(q_x dB'/\pi)_{b3}$	E_{b3}
0.103	50	1.5	0.032614000	0.52				
0.413	50	3.0	0.121933969	7				
1.148	50	5.0	0.254616366	26				
4.593	50	10.0	0.409608906	46	0.812446482	36	0.812446482	17
0.207	100	1.5	0.064540495	4				
0.827	100	3.0	0.208673872	37				
2.297	100	6.0	0.344052483	78	0.650717012	21		
9.186	100	10.0	0.450735867	98	0.900365990	91	1.34754225	79

S.II. Band gap opening, non parabolicity and numerical degradation in gapless bilayer graphene

The Hamiltonian that describes bilayer graphene with band gap opening and non parabolicity is given as,[3]

$$H = \begin{pmatrix} V_1 & \pi & t & 0 \\ \pi^\dagger & V_1 & 0 & 0 \\ t & 0 & V_2 & \pi^\dagger \\ 0 & 0 & \pi & V_2 \end{pmatrix}, \tag{S.8}$$

where $\pi = v_F(p_x + ip_y)$, $p_{x,y} = -i\hbar\partial_{x,y}$ represents the momentum operator, v_F is the Fermi velocity and $t \approx 390$ meV describes the interlayer coupling. V_1 and V_2 represent the electrostatic potential at the top and bottom layers, respectively. In fact, the difference

between these potentials is the band gap, $E_g = V_1 - V_2$, induced by gating. With this Hamiltonian and the fundamentals of the transfer matrix approach it is possible to obtain the transmission properties of bilayer graphene superlattices. The particular results for BGSBs are presented in Fig. S3. As we can notice the Fano profile is deformed as the band gap increases and it is practically destroyed for a critical band gap.

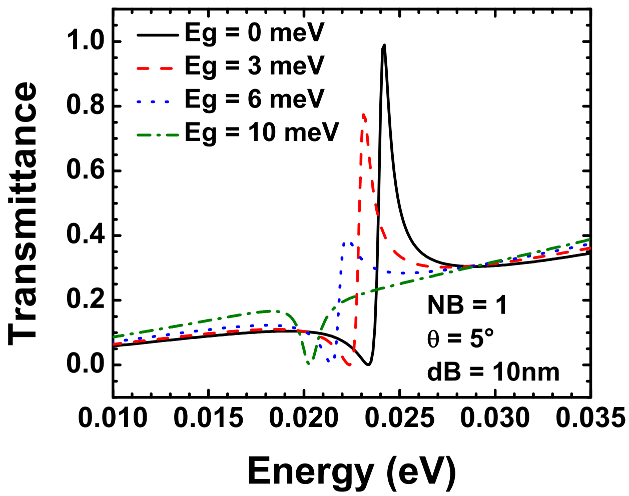

Fig. S3: Evolution of Fano resonances for various band gaps in bilayer graphene single barriers. As we can notice as the band gap increases the Fano resonance red shifts. Furthermore, the Fano profile is practically lost after a band gap of 10 meV. The band gap is given as $E_g = V_1 - V_2$. In our case V_1 varies, while V_2 remains fixed at 50 meV.

Non parabolicity is relevant at energies close to the interlayer coupling $t = 390$ meV. So, in order to see how this effect modify the Fano profile it is necessary to shift the Fano resonance to energies near 390 meV. By systematically increasing the barrier height it is possible to shift the Fano resonance to those energies. Our concrete results for double barriers are shown in Fig. S4. It seems that non parabolicity is a quite relevant effect, however the numerical degradation that is presented with the transfer matrix and the four band Hamiltonian, eq. (S.8), impede us to have a definitive answer. To solve numerical instabilities a full treatment with the hybrid matrix method is needed as in the case of the two band Hamiltonian.[4]

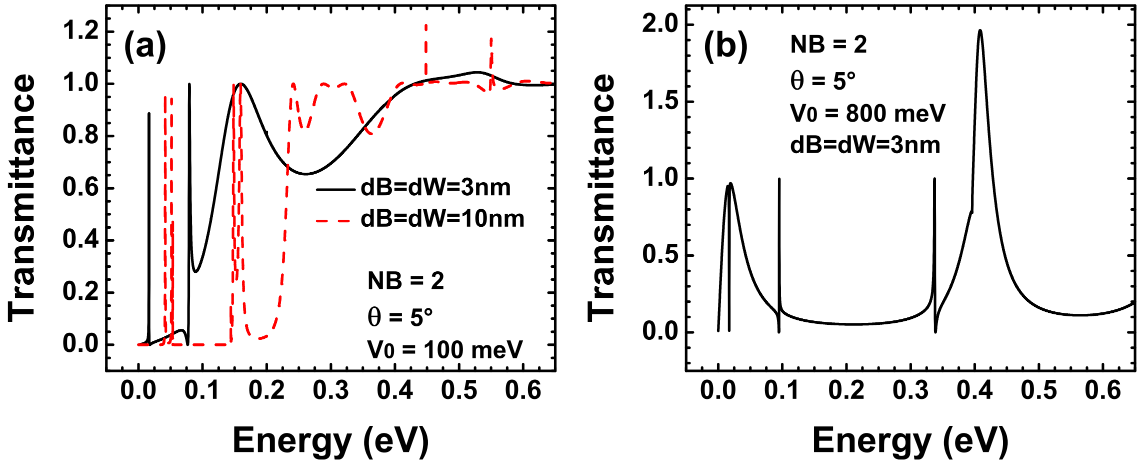

Fig. S4: Transmittance as a function of the energy obtained with the transfer matrix method for the case of bilayer graphene double barriers. As we can notice the transmittance presents unacceptable values in some energy regions, values above 1, due to the numerical instabilities associated with the transfer matrix approach. Actually, numerical degradation is larger in the region at which non parabolicity is relevant, 390 meV, see the figure at the right. Here, we are considering gapless double barriers with different heights: (a) $V_1 = V_2 = 100$ meV and (b) $V_1 = V_2 = 800$ meV. In (a) the solid-black line and the dashed-red curves correspond to $dB = dW = 3$ nm and $dB = dW = 10$ nm, respectively, while in (b) we only consider the case of $dB = dW = 3$ nm. In both figures the angle of incidence is $\theta = 5°$.

Supplementary References

1. N. Gu, M. Rudner and L. Levitov, "Chirality-assisted electronic cloaking of confined states in bilayer graphene," Phys. Rev. Lett. **107**, 156603 (2011).

2. P. Markos and C. M. Soukoulis, "Wave Propagation: From Electrons to Photonic Crystals and Left-Handed Materials," Princeton University Press, New Jersey, 2008.

3. E. McCann and M. Koshino, "The electronic properties of bilayer graphene," Rep. Prog. Phys. **76**, 056503 (2013).

4. J. A. Briones-Torres, R. Pernas-Salomón, R. Pérez-Álvarez and I. Rodríguez-Vargas, "Hybrid matrix method for stable numerical analysis of the propagation of Dirac electrons in gapless bilayer graphene," Superlattice. Microst. **93**, 186-201 (2016).
